# Inter-Annual and Seasonal Variations of Water Quality and Trophic Status of a Reservoir with Fluctuating Monsoon Precipitation

**DOI:** 10.3390/ijerph18168499

**Published:** 2021-08-11

**Authors:** Ye-eun Moon, Hyun-su Kim

**Affiliations:** Department of Earth & Environmental Sciences, Jeonbuk National University, Jeonju 54896, Korea; yeeun9149@jbnu.ac.kr

**Keywords:** reservoir, trophic state index, nutrients, organic matter, drainage ratio

## Abstract

Long-term evaluation from 2000 to 2020 of a temperate reservoir indicated that water quality and trophic status were not critically controlled by the inter-annual dynamics of monsoon precipitation. The fluctuation of annual concentrations of TP, TSS, and EC did not always correlate with the variation of precipitation. BOD and COD demonstrated monotonically increasing trends with Sen’s slope of 0.02 and 0.01, respectively, while Chl-*a* manifested a decreasing trend (slope = −0.23). The increases of different magnitudes in the levels of TP, TSS, and Chl-*a* in the monsoon and the early post-monsoon periods were observed in the drought, flood, and normal years. The drought years showed distinct seasonal variations in many parameters, while those in the flood and the normal years were very similar. Pearson correlation and empirical regression analyses resulted in weaker correlation between water quality and hydrological parameters than other reservoirs, which, along with low impact of precipitation, was attributed to the low drainage ratio (28.8) of the study area. BOD/COD ratios were higher than 0.5 in the reservoir, indicating the presence of a significant fraction of biodegradable organic matter. The ratio increased in the longitudinal flow direction (>50% in the drought years), implying the possibility of autochthonous sources of organic matter. TSID evaluation resulted in variation of limiting factors not related to the fluctuation of annual precipitation. The loadings of the significant principal components were very similar among the different precipitation groups, supporting the other findings that annual precipitation governed by monsoon intensity would not solely control the water quality dynamics of the study area.

## 1. Introduction

Global water use has increased almost six times over the last 100 years, and is expected to grow around the world in the near future [1]. Although annual precipitation has also increased in the last century, the rate of increase was not high enough to keep up with the increasing demand, causing water scarcity in many parts of the world [2]. The imbalance between demand and supply has become a serious problem, especially in East Asia, including South Korea, where more than a half of annual rainfall is concentrated over a couple of months in the summer [3]. Many artificial dams have been built by blocking rivers to create reservoirs for the storage and stable supply of water throughout the year, and now there are approximately 19,000 reservoirs in Korea [4]. The reservoirs are the central component of the national water supply system. As many of the reservoirs are utilized as drinking water sources, water quality management is one of the primary goals of reservoir management [5,6].

Despite the importance of maintaining good water quality, the innate physical characteristics and their variabilities of reservoirs complicate efficient reservoir management [7,8,9,10,11]. Artificial reservoirs are known to have large spatiotemporal variation of water quality parameters, such as nutrients (total nitrogen (TN) and total phosphorus (TP)), chlorophyll-*a* (Chl-*a*), suspended solids (SS), and water clarity represented by Secchi depth (SD) due to the fluctuating traits of hydraulic characteristics in space and time [12]. Longitudinal zonation in the flow direction from the headwater to the dam gives rise to spatial heterogeneity in water quality [13,14,15,16]. In addition, fluctuating inflow and discharge controlled by seasonally changing precipitation and water demand, respectively, create differences in hydraulic retention time (HRT), water budget, and input and enrichment of pollutants with a watershed origin [8,9]. Many reservoirs indeed are suffering from water quality problems, especially due to elevated nutrient concentrations, such as nitrogen (N) and phosphorus (P), which result in a eutrophic condition, frequently leading to the occurrence of harmful algal blooms that produce many associated problems [17,18,19].

Controlling the enrichment of nutrients, especially P in many freshwater reservoirs, requires an understanding of how they are introduced and circulated in the diverse components of an aquatic ecosystem. The most important driver of the changes in hydraulic properties and pollutant input is distribution and intensity of precipitation, and highly variable precipitation can create uncertainties in water quality management. In this regard, the reservoirs in Korea have the potential to be seriously affected by the summer monsoon that occurs in July and August [20,21,22,23]. Intense monsoon increases inflow of water containing excessive concentration of nutrients, and decreases hydraulic retention time (HRT) to control nutrient loading and algal growth in the reservoir [14,24]. Lack of precipitation in the monsoon season also affects physicochemical conditions of a water body, as it affects runoff, water body flow, and water levels, which have the potential to control the production of cyanobacterial blooms that could be linked to higher algal productivity [25].

In the long-term assessment of reservoirs, little effort has been invested in understanding the input and cycling of organic matter (OM) in the artificial ecosystem [26,27]. OM has a significant implication in water quality management, as some types are resistant to biodegradation and persist in a lentic water body [28]. Refractory OM can act as a precursor of chlorinated disinfection byproducts (DBPs) in water treatment or increase mobility of heavy metals [29,30,31]. OM in a reservoir can have either an allochthonous or autochthonous source. Allochthonous OM enters the water primarily in the form of runoff during precipitation events [32,33], while autochthonous OM is produced in situ through photosynthesis by phytoplankton and microorganisms [34]. Allochthonous OM contains many humic components with aromatic ring structures [35] and constitutes a major refractory component of natural organic materials [36], while autochthonous OM frequently consists of biodegradable compounds such as carbohydrates, proteins, and amino acids [34]. Therefore, the concentration and refractory nature of OM have the potential to be affected by monsoon rain distribution, as it will affect the relative contribution of autochthonous and allochthonous sources.

The purpose of this study is to evaluate the interaction between water quality variables and hydrological characteristics, including the inter-annual variation of monsoon precipitation, by analyzing long-term monitoring data of the Okjeong Reservoir, a large multi-purpose reservoir located in Korea, where two local governments are in conflict over the development of the reservoir’s water surface. Although it is necessary to identify the factors governing water quality change to assess the impact of development, long-term evaluation of the dynamics of water quality and trophic status has never been attempted. Traditional assessment tools such as TSID evaluation, empirical regression analysis, and multivariate statistical analysis were utilized, in addition to evaluating long-term concentration changes of various water quality parameters in response to changing annual precipitation. Physical characteristics of the reservoir and the watershed, as well as spatiotemporal changes of organic parameters and their ratios, were also evaluated in an effort to identify the intensity of the impact of precipitation on reservoir water quality dynamics, and sources and refractory nature of the OM in the reservoir.

## 2. Materials and Methods

### 2.1. Study Area

**Figure 1:**
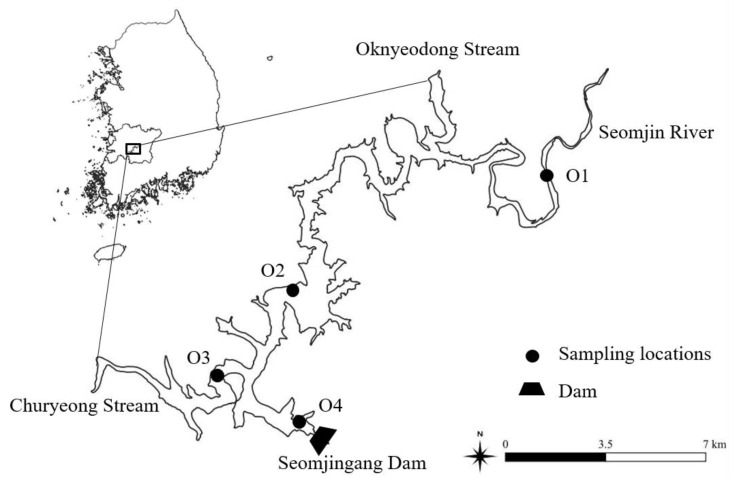
The map of the study area with sampling locations in the Okjeong Reservoir.

The study area, the Okjeong Reservoir, is located at 35°32′27″–35°37′53″ N and 127°01′36″–127°12′20″ E in Jeonbuk Province, South Korea, and at the uppermost reach of the Seomjin River (Figure 1). The reservoir was initially created when Unam Dam was constructed upstream of the river in 1929 to supply water to the agricultural area in the watershed of the nearby Dongjin River. Seomjingang Dam, the first multi-purpose dam in South Korea, was built in 1965 about 2 km downstream of Unam Dam to increase the reservoir’s storage capacity for the stable supply of water to the neighboring municipalities for agricultural irrigation, domestic uses, and power generation. The dam stands 64 m high and 344.2 m long. The reservoir has the surface area of 26.5 km^2^, and is the 10th-largest reservoir in the nation in terms of storage capacity, with total storage capacity of 4.66 × 10^8^ m^3^. The area of watershed is 763 km^2^ constituting 15.6% of the Seomjin River watershed, and is subdivided into four municipalities, Imsil-gun, Jinan-gun, Sunchang-gun, and Jeongeup-si. Jeongeup-si proposed a development plan for the water surface, and has since been in conflict with Imsil-gun, which raised the concern of water quality degradation. The land-use pattern includes 68.5% as mountainous-forested terrain in the watershed, while farmland accounts for nearly 17.6%, followed by grassland, undeveloped areas, and urbanized areas, each occupying 5.9%, 2.3%, and 2.1%, respectively. In addition to the mainstream Seomjin River, the Oknyeodong Stream and Churyeong Stream are minor tributaries of the reservoir.

### 2.2. Water Quality and Hydrological and Meteorological Data

The monthly data for the surface water quality parameters from 2000 to 2020 were obtained from the national water quality measurement network operated by the Ministry of Environment [37]. This investigation included four study sites in the Okjeong Reservoir, where monthly measurement of water quality parameters are made (Figure 1). Site O1 was located at the inlet of Seomjin River, O2 and O3 in the downstream half of the reservoir, and O4 in the deepest part of the reservoir near the dam. The parameters collected for the analyses were water temperature (WT), TN, TP, total dissolved nitrogen (TDN), total dissolved phosphorus (TDP), Chl-*a*, SD, biochemical oxygen demand (BOD), chemical oxygen demand (COD), and electrical conductivity (EC). Total particulate phosphorus (TPP) and nitrogen (TPN) were obtained by subtracting TDP and TDN from TP and TN, respectively. Monthly precipitation data were obtained from the Korean Meteorological Administration, and the data measured at the weather station located in the study area (Imsil station) were used for analysis. The information on the reservoir’s operation, such as inflow, outflow, and monthly volume of water storage, was collected from the Korean Water Resource Corporation.

### 2.3. Trophic State Index and Its Deviation

TSIs for Chl-*a*, TP, and SD of the reservoir were calculated using the methods proposed by Carlson [38]. The Carlson’s TSI (TSI_C_) can be obtained by calculating the arithmetic average of the three TSIs. TSI has the advantage of presenting the trophic state on a continuous numeric scale. Waters with TSIs less than 40 are categorized as oligotrophic, 40 to 50 mesotrophic, 50 to 70 eutrophic, and higher than 70 hypereutrophic [39,40]. TSI_TN_ was also calculated using the method in the literature [39]. The equations used for calculation of TSIs were:

(1) $${\mathrm{TSI}}_{\mathrm{Chl}-a}=10\times \left[-\frac{2.04-0.68\ln\mathrm{Chl}-a}{\ln2}\right]$$

(2) $${\mathrm{TSI}}_{\mathrm{TP}}=10\times \left[6-\ln\left(\frac{48}{\mathrm{TP}}\right)/\ln2\right]$$

(3) $${\mathrm{TSI}}_{\mathrm{SD}\;}=10\times \left[6-\ln\mathrm{SD}/\ln2\right]$$

(4) $${\mathrm{TSI}}_{\mathrm{TN}\;}=10\times \left[6-\ln\left(\frac{1.47}{\mathrm{TN}}\right)/\ln2\right]$$

As parameters used in Carlson’s TSI are to represent algal biomass in water, TSIs of different variables should be identical if biological productivity is limited by *p* and turbidity of water results from phytoplankton particles. In practice, however, TSIs of different parameters are frequently different from each other, and it was proposed that differences in numerical magnitudes of TSI_Chl_, TSI_TP_, and TSI_SD_ can be used to identify different limiting conditions in an aquatic ecosystem [7,41,42]. A simple two-dimensional graphical method in which TSI_Chl_—TSI_SD_ values are plotted against TSI_Chl_—TSI_TP_ was proposed for identification of relationships between the trophic state variables [41,43]; this method was utilized in this study. This approach can be used to interpret deviations caused by nutrient limitation, non-algal turbidity, zooplankton grazing, and water color imparted by dissolved constituents.

### 2.4. Statistical Analysis

Pearson correlation analysis, a univariate statistical analysis widely applied to evaluate relationships between two variables in a data set by measuring the strength, direction, and probability of the linear association between two interval or ratio variables, was applied to identify one-to-one statistical dependence between the variables studied. He correlation coefficient (*r*), the numerical value of which ranges from −1 to 1, was the statistic used in the analysis. While *r* values close to 1 and −1 indicate strong positive and negative relationships, respectively, no correlation exists if *r* = 0 at a significance level of *p* < 0.05.

Major hydrological and biogeochemical processes affecting the trophic state of the reservoir were elucidated using principal component analysis (PCA). PCA is a multivariate statistical analysis that is used to reduce the dimension of a large number of data into a small number of factors or principal components (PCs), which are used to explain the observed spatiotemporal variation of various trophic states and water quality parameters. Two correlation matrices for each precipitation group were used to extract PCs. The input parameters of the PCA were measured values of water quality and reservoir morphology, meteorological data, and calculated values of TSIs. They were first standardized to z-scores that had a mean of 0 and standard deviation of 1. The standardization was performed to give equal weight to each variable, to prevent results from being influenced strongly by variables of great magnitudes [44]. The components with eigenvalues greater than 1 were selected and rotated iteratively by the varimax method to maximize the variance to find factors that could be easily explained by the biological and hydrogeochemical processes [45]. Kaiser–Meyer–Olkin (KMO) and Bartlett’s tests were performed to check the suitability of the analysis.

A regression analysis for empirical modeling was performed after logarithmically converting (base 10) the concentrations of TN (mg/L), TP (mg/L), Chl-*a* (μg/L), and SD (m) to promote homogeneity of variance. The relationships among nutrients, Chl-*a*, and SD were explored using simple linear regression models, with Chl-*a* as the dependent variable of TN and TP, and the SD of TN, TP, and Chl-*a*. Since a 95% quantile regression model can be used to estimate the rates of change for functions close to the upper boundary of a conditional distribution of responses [46], the nutrient—Chl-*a* relationships based on 95% quantile regression were implemented to explore a 95% maximum response of Chl-*a* to nutrient levels, and the same operation was also conducted for SD. All the statistical analyses were conducted with PASW Statistics 18 software (SPSS Inc., Chicago, IL, USA).

A trend analysis was performed to identify the temporal trend of long-term data used in this study. The Mann–Kendall (MK) test, a non-parametric analysis tool, was used as the trend analysis method [47]. The trend was identified by verifying the significance using the *p*-value calculated through the MK test. The null hypothesis that there was no trend was accepted if the *p*-value for the significance level (*α* = 0.05) was less than 0.05 on either side of the 95% confidence interval. If the *p*-value was greater than 0.05, the null hypothesis was rejected, and the alternative hypothesis was accepted [48]. Then, an increasing temporal trend was indicated when the S-value had a positive value, and a decreasing trend when the value was negative [48]. Sen’s slope analysis was used to visually represent the trend. In this study, XLSTAT was used for the MK test.

## 3. Results and Discussion

### 3.1. Inter-Annual Precipitation and Dam Hydrology

**Figure 2:**
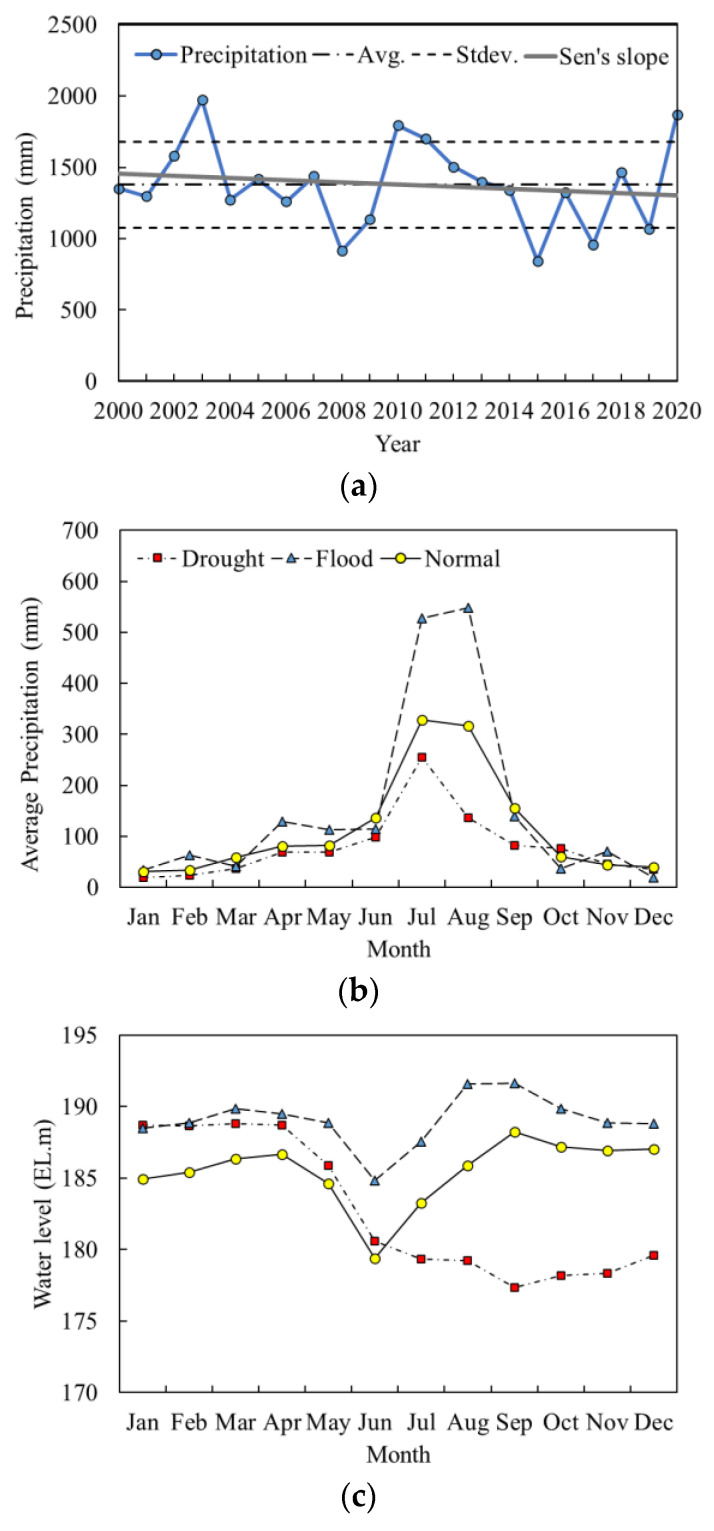
Temporal variations of key hydrological parameters. (**a**) Inter-annual variation of total annual precipitation, and monthly variation of (**b**) precipitation and (**c**) water levels of the Okjeong Reservoir in the drought, flood, and normal precipitation years.

The annual precipitation data from 2000 to 2020 depicted in Figure 2 demonstrated significant inter-annual variation. Average annual precipitation over the study period was 1377.2 mm with a standard deviation of 301.0 mm. A maximum precipitation of 1974.2 mm was recorded in 2003, and a minimum of 843.1 mm in 2015. In this study, the years in which the annual precipitation was within one standard deviation of the average precipitation were grouped as a normal precipitation year group, and those with annual precipitations more than one standard deviation above and below the average precipitation were classified as flood year and drought year groups, respectively. Based on this classification scheme, the years 2008, 2015, 2017, and 2019, when annual precipitations were 915.3, 843.1, 958.3, and 1069.7 mm, respectively, were assigned to the drought group; and 2003, 2010, 2011, and 2020, with precipitations of 1974.2, 1795.1, 1700.0, and 1869.8 mm, respectively, were classified as the flood group. The results of the MK trend analysis indicated a decreasing trend of precipitation with time, although the slope calculated was not statistically significant (Table 1).

The inter-annual variation of yearly precipitation turned out to be directly controlled by the intensity of summer monsoon, as evidenced by the monthly distribution of rainfall in the normal, flood, and drought groups (Figure 2b). Average monthly precipitations of the flood years in July and August were 527.4 and 547.7 mm, respectively, while corresponding values of the drought years were 255.7 and 136.8 mm. Based on the monthly precipitation characteristics, January through June were defined as the pre-monsoon period, July and August as monsoon, and September through December as post-monsoon.

Changing intensity of the summer monsoon also affected reservoir operation during the study period. Similar water levels and their monthly variations were observed in the pre-monsoon period (Figure 2c). The rapid decrease in water levels in June was due to the discharge of water from the reservoir to prevent flood damage from intense rainfall during the monsoon period. A pronounced difference in the trends of water level changes among the groups was observed during the monsoon period. While rapid increases in water level were observed in the flood and normal years, a gradual decrease was observed in the drought group. The enlarged gaps between the drought years and the other groups were maintained for the duration of the post-monsoon period. Monthly changes in the inflow followed precipitation patterns in the normal and flood years, while a significant increase in discharge occurred one or two months after inflow (Figure S1). On the contrary, the discharge increased earlier than the inflow in the case of drought years, although the values were not as high as in the other two groups.

### 3.2. Inter-Annual Variations in Water Quality Parameters and Their Trends

**Figure 3:**
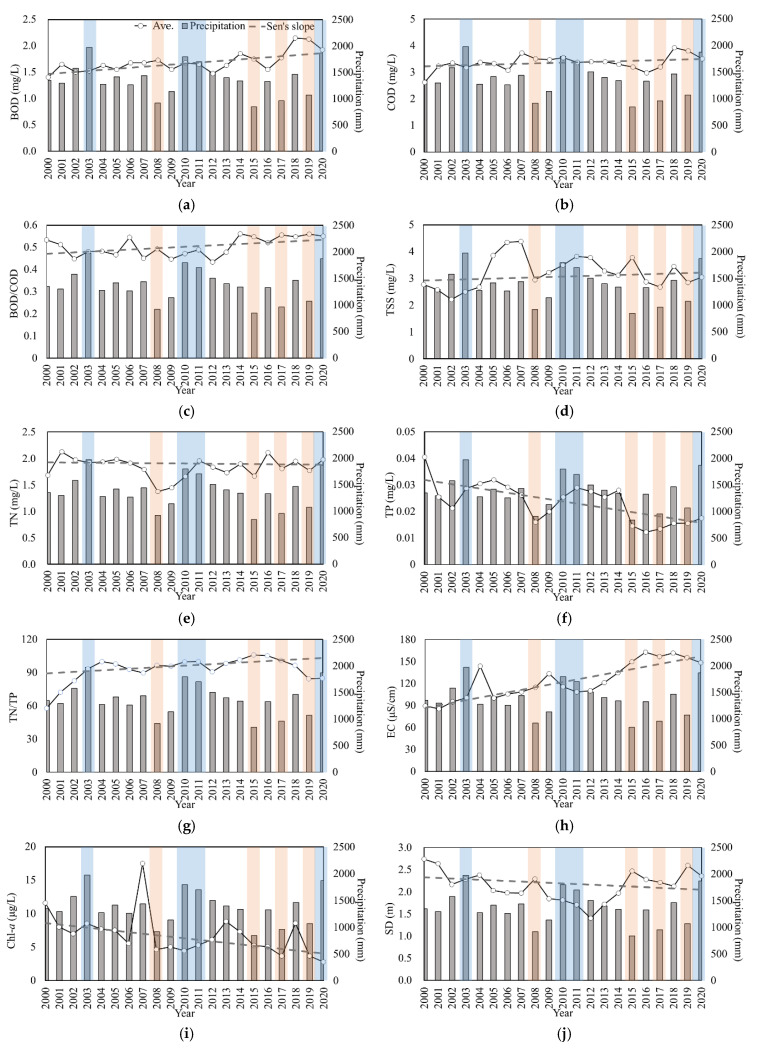
Yearly variation of water quality parameters and the results of the long-term trend analysis: (**a**) BOD, (**b**) COD, (**c**) BOD/COD, (**d**) TSS, (**e**) TN, (**f**) TP, (**g**) TN/TP, (**h**) EC, (**i**) Chl-*a*, and (**j**) SD. Bar graphs in the background display the total annual precipitation. The shaded areas in blue and pink represent the flood and drought years, respectively.

Temporal distributions of average annual concentrations of water quality parameters including nutrients (TN, TP), organic pollutants (COD, BOD), suspended solids (TSS), and algal productivity (Chl-*a*) demonstrated pronounced differences in their responses to the inter-annual variation of total precipitations (Figure 3). Nutrient concentrations showed rather contrasting temporal behavior, especially in the second half of the investigation period. Although average annual TN concentrations in three of the four flood years showed an increase from the year before, the differences were not huge, and the values were frequently lower than the years with normal precipitation a few years prior. Rather, the changes were more pronounced in the drought years showing significant decreases from the year before. In the second half, TN maintained relatively constant concentrations after the rapid increase from a drought year (2008) to the flood years (2010 and 2011) when a similar increasing trend was observed with TP. TP concentration, however, dropped from 0.028 mg/L in 2014 to 0.015 mg/L in 2015, when the lowest annual precipitation was recorded in the study period, and remained low in the last six years. Although the results of the MK trend analysis indicated no trend in TN and a decreasing trend in TP, TP concentrations showed a stronger association with the fluctuation of inter-annual precipitation with increase in the flood years and decrease in the drought in the first half, gradual decrease with declining precipitation from 2011 to 2014, and lower values in the interval from 2015 to 2019 when three drought years occurred. The N/P ratio (TN/TP) remained much higher than 20, the threshold value frequently utilized to determine a P-limited condition [49]. In the first half of this study, average N/P values remained relatively constant around 95, then increased monotonically until 2015 to 106 due to the decreasing trend of TP, especially toward the end of the study period.

Chl-*a* showed a generally decreasing trend without consistent relationships with precipitation, and recorded the lowest average concentration of 2.8 mg/m^3^ in 2020. The years in which significant increases in Chl-*a* levels were recorded were 2007, 2013, and 2018, all belonging to the normal precipitation group. The declining patterns were more evident in the first and last 6-year intervals, when a larger fluctuation of precipitation was observed. It was reported that a valid reason for this decline could be the successive increase in annual rainfall, which could have washed out most of the algal Chl-*a* [50].

Although temporal distribution of annual concentrations appeared to indicate an increasing trend in TSS concentrations, a significant increase and decrease in the flood and the drought years, respectively, were not always observed, and maximum annual concentrations were observed in the years that were in the normal precipitation group. SD marked the maximum value in the beginning of the investigation, and a nearly monotonic decrease was observed until 2012, after which it increased until 2015 and remained relatively constant. SD appeared to have stronger relations with the drought conditions, as evidenced by the increase in 2008 and generally high values from 2015 to 2019.

BOD and COD showed generally increasing trends, especially BOD. The flood and drought years did not show a consistent increase or decrease from the prior year for either parameter. The most prominent feature of temporal distributions of the organic parameters was a significant increase toward the end of the study period, with the gradient higher than the first two-thirds. The fact that BOD and COD maintained mostly increasing trends without strong association with precipitation change may indicate that their increases might have been caused by the changes in anthropogenic activities occurring in the surrounding watershed [7,51]. Indeed, a minor increase in industrial pollution sources was reported over the period from 2012 to 2016, with a concurrent decrease in farming activities [52]. EC showed an increasing trend during the study period, and the most significant variation with precipitation, a decrease in the flood years, and an increase in the drought years, which represented the effects of dilution and concentration, respectively [53].

### 3.3. Seasonal Variations with Different Monsoon Intensity

**Figure 4:**
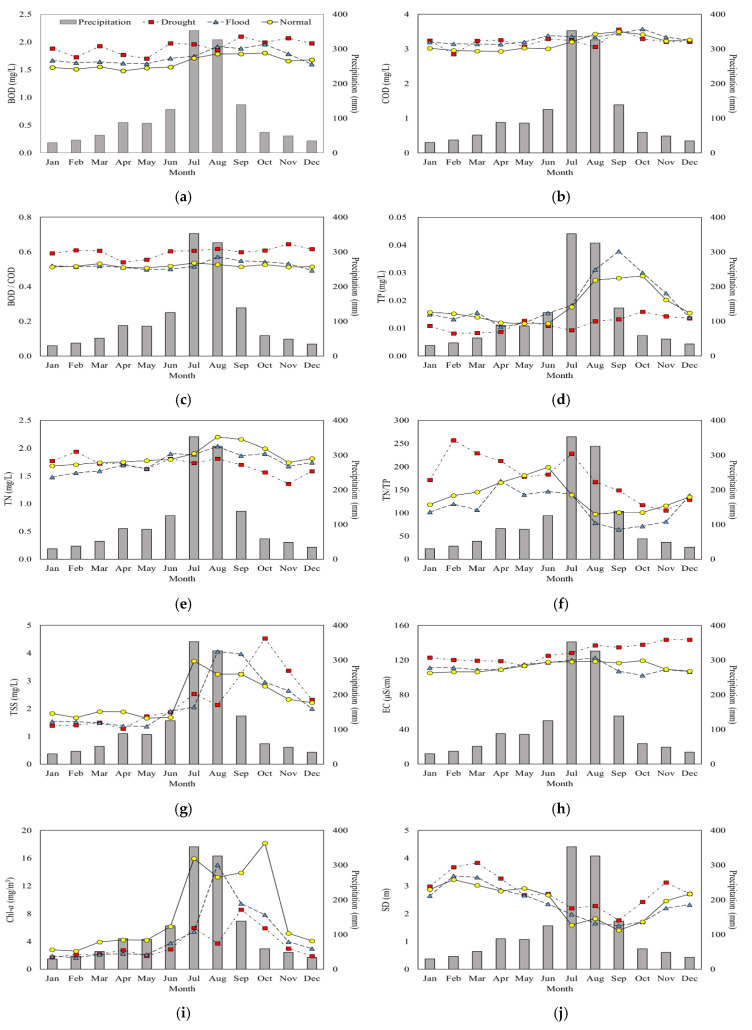
Monthly variation of water quality parameters in the drought, flood, and normal years: (**a**) BOD, (**b**) COD, (**c**) BOD/COD, (**d**) TP, (**e**) TN, (**f**) TN/TP, (**g**) TSS, (**h**) EC, (**i**) Chl-*a*, and (**j**) SD. Bar graphs in the background display the average monthly precipitation over the 20-year study period.

Monthly variation of TP was much greater than that of TN under different monsoon regimes, although the lowest concentrations of both parameters were observed in the drought group for most of the year (Figure 4). TP concentrations in the monsoon period appeared to be closely related to the intensity of rainfall, with a significant increase in the monsoon and early post-monsoon periods of the flood and the normal years, with higher levels observed in the flood. In the drought years, however, only a slight increase in concentrations to October was observed in the corresponding period. Seasonal variation of TN concentrations did not show a pronounced difference among the groups in terms of concentration levels and their temporal trends. Only a slight increase was observed in the monsoon season in all of the groups, and the concentrations of the normal and the flood years were very similar to each other throughout the year, while an observable difference was recorded in the drought years from July to November. N/P ratios were generally higher in the pre-monsoon period of each group, and the values in each month were highest in the drought years, followed by the normal and the flood years, indicating the impact of precipitation on the ratio due to the association of TP with precipitation as observed in other reservoirs in Korea [7,54]. The contrasting behavior of the nutrients during the monsoon period may be due to the differences in physical states of materials that constitute each nutrient. Calculation of the fraction of TN occupied by TDN resulted in nearly constant value of 90% throughout the investigation period. TP, however, turned out to be composed of approximately 60% TPP and 40% TDP, indicating the possibility that TN may not be subject to sedimentation or flushing due to increased flow rates.

Monthly variations of Chl-*a* concentrations were generally similar to that of TP, although Chl-*a* concentrations from the monsoon to early post-monsoon periods (July to October) were higher in the normal years than the flood years. High concentrations were maintained from July to October in the normal years, while a rapid increase and decrease were observed before and after August in the flood years. The notable difference between TP and Chl-*a* levels in the summer between the flood and the normal years seemed to indicate that the factors influencing the concentrations of these parameters were different. TP seemed to be mostly controlled by increased inflow due to intense rainfall. As for the lower Chl-*a* levels in the flood years, this might have been caused by a flushing effect during the season that did not allow the phytoplankton to achieve a high concentration.

There have been contrasting arguments over the impact of precipitation on phytoplankton communities in the reservoirs. Some claimed that increases in annual precipitation and extreme precipitation events would increase harmful algal blooms due to enhanced input of nutrients from the watershed [55,56], while others argued this could be balanced by greater flushing as a result of intense rainfall preventing bloom formation [57]. It was also reported that reduced water depth induced by extended drought conditions fostered phytoplankton growth [25]. The results obtained in this study implied that phytoplankton might not be given enough time required to achieve growth in the reservoir due to significantly reduced water retention time [58], as indicated by the inflow and outflow in the monsoon season (Figure S1). Despite the fact that TP did not vary much from July to October in the drought years, an appreciable increase in Chl-*a* level was observed in the period, indicating that phytoplankton growth might have been controlled in the drought years by factors other than nutrient concentrations, such as physicochemical conditions of water, including temperature and stability of the water body, and a decrease in water depth [25,59,60,61]. The months in which maximum concentrations were recorded were different among the groups. The maximum Chl-*a* occurred earlier in the flood years in August (15.0 mg/m^3^), while the maximum concentration of 18.2 mg/m^3^ was recorded in October in the normal years. The drought years’ level was similar to the flood years, except for the month of August. TSS concentrations showed similar overall profiles to those of TP and Chl-*a*, especially from January to August. The drought years, however, showed a very rapid increase from August to October in comparison with the other two groups, although this increasing trend was reversed with a similar absolute value of the slope until December. SD values were higher in the drought years and displayed very similar monthly distribution among the groups, with minor differences in each month. SD showed a mostly inverse relationship with precipitation, which corresponded to the results obtained in the study on multiple lakes in Wisconsin, USA [62].

COD concentrations did not show an appreciable difference among different groups in each month. Although slight increases in concentrations were observed in each group from the beginning of the year to September or October, the difference was very minor (<0.4 mg/L). As can be seen in Figure 3, COD also did not show much of a relationship with precipitation in the inter-annual comparison with precipitation. No change in concentration over the time scale of a year indicated that there was a very insignificant correlation, if not none, that existed between precipitation and COD in the study area. In the case of BOD, nearly identical patterns to those observed in COD were identified in the normal and the flood years. The drought years, however, recorded monthly concentrations that were higher than the other groups for the most part of the year, except for August and October. BOD/COD ratios mostly remained in the range of 0.5 to 0.6, and did not demonstrate noticeable seasonal trends. The values in the drought years ranged from 0.54 in April to 0.64 in November, with an average value of 0.6, and were higher than the other two groups throughout the year. The flood and the normal years demonstrated practically identical values and trends, with monthly values of the ratio very close to 0.5. The monthly changes in organic parameter concentrations clearly indicated contrasting behavior to those that were sensitively affected by precipitation, such as TP, TSS, and Chl-*a*.

EC was higher in the drought period than the other two periods, and the practically identical values were observed in the flood and the normal years, as with the BOD/COD ratio. The higher value in the drought years can be attributed to low precipitation, as evidenced by the increasing trend of differences from the monsoon season.

### 3.4. Spatial Variations with Different Monsoon Intensity

**Figure 5:**
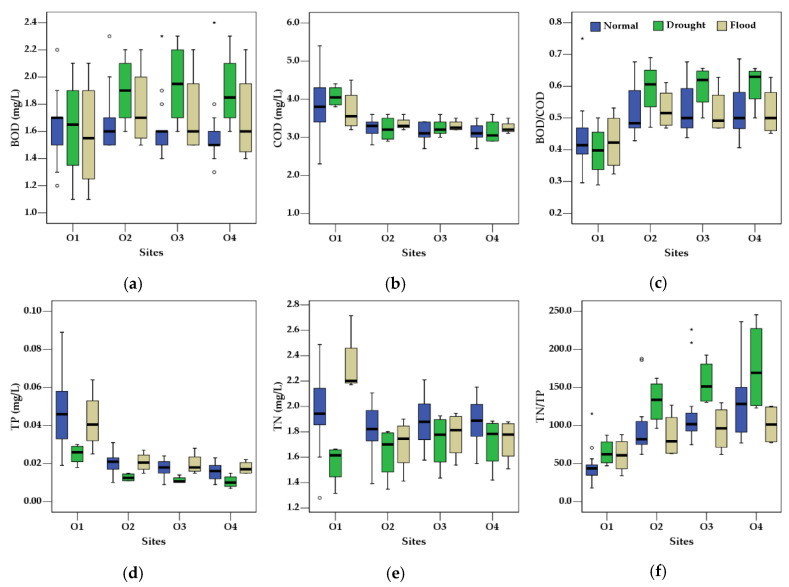

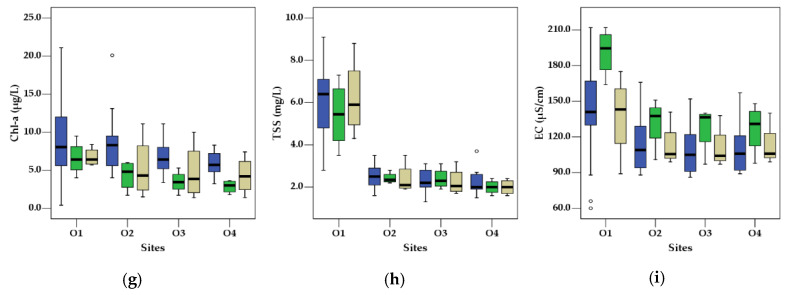
Spatial variations of water quality parameters in the study area: (**a**) BOD, (**b**) COD, (**c**) BOD/COD, (**d**) TP, (**e**) TN, (**f**) TN/TP, (**g**) Chl-*a*, (**h**) TSS, and (**i**) EC.

The two parameters that represented organic pollutant concentrations in the water, BOD and COD, showed contrasting longitudinal trends in the sampling locations (Figure 5). COD at site O1 was appreciably higher than the other sites consistently in all of the year groups. Gradually decreasing trends of concentrations were observed from sites O2 to O4, and the average concentrations at each location among the three groups were very similar, with statistically insignificant differences (*p* > 0.05). On the contrary, the longitudinal trends of BOD levels with locations were quite different in each precipitation group. The normal years showed monotonically decreasing concentrations from O1 to O4, as observed in the case of COD, and the flood years demonstrated rather constant concentrations in each location. Overall concentration changes in these two groups were minor at different locations, although the flood years demonstrated a much higher variation. The drought years showed a significantly different distribution from the other groups. A significant increase from O1 to O2 was observed, which stabilized in O3 and O4. The BOD/COD ratio was always higher for the sampling locations within the reservoir than the one for the inlet in the three precipitation groups. Although relatively similar values were observed in the reservoir inlet, those from O2 to O4 were always higher in the drought years, while the flood and the normal years showed very similar values in each group of around 0.5. A temporally stable BOD/COD ratio and its increasing trend in the longitudinal flow direction of the reservoir may provide qualitative information on how the organic material was introduced to the water body.

Based on the innate characteristics of BOD and COD measurements, the BOD/COD ratio has been widely utilized as an indicator of recalcitrance of organic matter to biodegradation [63]. A low ratio indicates an increase in non-biodegradable organic matter flowing into rivers and lakes from the watershed [64]. It was reported in a statistical analysis that the average value of the ratio calculated from 81 reservoirs in Korea was 0.42 [65]. The average BOD/COD ratio in the reservoirs and the rivers reported in another study were 0.31 and 0.48, respectively [66], indicating that the values obtained in this study were significantly higher than the ambient values commonly encountered in nature, and a significant fraction of the OM in Okjeong Reservoir was biodegradable material. OM in a reservoir may have either an allochthonous or autochthonous source. Allochthonous OM enters the water primarily in the form of runoff during a precipitation event [32,33], while autochthonous OM is produced in situ through photosynthetic and microbial activities [34]. Allochthonous OM contains many humic components with aromatic ring structures [35] and constitutes a major refractory component of natural organic materials [36], while autochthonous OM frequently consists of biodegradable compounds such as carbohydrates, proteins, and amino acids [34]. An analysis of fluorescence characteristics of natural organic substances present in lakes and rivers clearly showed protein-based fluorescence characteristics constituting biodegradable organic substances at the point of phytoplankton and microbial activity [28]. The high BOD/COD ratio in general, inert temporal distribution to precipitation, and increase in biodegradable fraction in the flow direction observed in the current study insinuated the possibility that OM in the Okjeong Reservoir was significantly contributed by internal sources of OM.

The location of the study area in the Seomjin River watershed also supported the possibility of higher-than-average autochthonous contribution. Park et al. [67] found in their investigation of the contribution of OM in the two reservoirs in South Han River watershed, Lake Chungju and Lake Paldang, that OM in Lake Paldang was mainly allochthonous, while a significant contribution of autochthonous organic carbon was identified in the case of Lake Chungju. While Lake Paldang is a river-type reservoir located in the downstream area of the river, Lake Chungju is a lake-type reservoir in the upstream Han River, which are characteristics shared with the Okjeong Reservoir.

TP concentrations showed a monotonic decrease in the longitudinal flow direction of the reservoir in all three precipitation groups. Similar concentrations were recorded at each location in the normal and the flood years, while concentrations in the drought years were always lower, with statistically significant differences at each location (*p* < 0.01). TN concentrations showed a large variation at site O1, and different concentration trends along the longitudinal direction among the three precipitation groups. A monotonic increase and decrease were observed in the drought and the flood years, respectively. Relatively constant concentrations were observed in the normal precipitation group, the concentrations of which at each location were higher than those of the other two groups. All three groups displayed a monotonically increasing trend of the N/*p* ratio within the reservoir. The highest ratio was observed in the drought group, with average values ranging from 47.0 at O1 to 245.4 at O4, followed by the normal and the flood year groups.

Chl-*a* concentrations showed minor longitudinal variation. The values were higher in the normal precipitation group, and those for the drought and the flood years were similar, although the flood years displayed a larger variation at each location. Monotonic decreases were observed in all three groups. TSS decreased significantly from site O1 to O2, from which a steady decrease within the reservoir was observed. Overall, noticeable differences among the three groups were not observed at each location. SD increased greatly at site O2, from which a continuous increase with a different gradient was observed in the three groups. The drought group was higher than the other two with statistically significant differences, while the normal and flood groups were quite similar. EC decreased with longitudinal distance from site O1, and was always higher in the drought group than the other two groups.

### 3.5. Correlations between Water Chemistry and Hydrology

Evaluation of the Pearson’s correlation coefficients between key hydrological and water quality parameters indicated mostly weak to no correlation between the flow regime and water quality parameters in the study period (Table 2). No water quality parameters showed any correlation with the precipitation. Inflow exhibited weak correlation with TSS and SD, with *r* values of 0.30 and 0.31, respectively. Outflow showed a weak correlation with SD. TSS showed a moderate positive correlation with TP (*r* = 0.5) and Chl-*a* (*r* = 0.4), and moderate negative correlation with SD (*r* = −0.5). TP showed a weak positive correlation with COD and TN (*r* = 0.3), and a weak negative correlation with SD (*r* = −0.4). TPP had a weak positive correlation with Chl-*a* (*r* = 0.4) and weak negative correlations with SD and EC (*r* = −0.3 for each). The N/P ratio displayed a moderately strong negative correlation with TP (*r* = −0.6) without any statistical relation with TN. The ratio’s correlation was stronger with TPP (*r* = −0.6) than TDP (*r* = −0.4), indicating that particulate *p* had more control of the ratio associated with strong intensity of monsoon rain. BOD showed a weak correlation with EC (*r* = 0.4), supporting the observation that BOD levels were higher in the drought years. COD showed a weak correlation with TSS (*r* = 0.4) and TDP (*r* = 0.3). BOD and COD did not show any appreciable correlations with other water quality and hydrological parameters.

### 3.6. Empirical Regression Analysis

An empirical regression analysis was applied to pre-monsoon, monsoon, post-monsoon and whole data of each precipitation group, and the results between the two nutrients and their ratio indicated seasonal discrepancies among the three groups (Table S1). TN and TP did not show any correlation (*R*^2^ < 0.10) in all the periods evaluated except for the pre-monsoon period of the drought years (*R*^2^ = 0.31). Correlations of TN/TP with individual nutrient concentrations turned out to be comparable between TN (*R*^2^ = 0.46) and TP (*R*^2^ = 0.40) in the drought years. Stronger correlations were observed in the pre- and post-monsoon periods than in the monsoon period. In the cases of the flood and the normal years, the correlation of the ratio was much higher with TP than TN. Very strong correlations were observed in the monsoon and post-monsoon periods of the flood years (*R*^2^ ≥ 0.77), while significant correlations were observed in all of the individual periods in the normal years (*R*^2^ ≥ 0.55). These observations supported what was observed in the monthly concentration profiles, and indicated the changing influence of precipitation on the dynamics of TP and TN/TP ratio with monsoon intensity.

**Figure 6:**
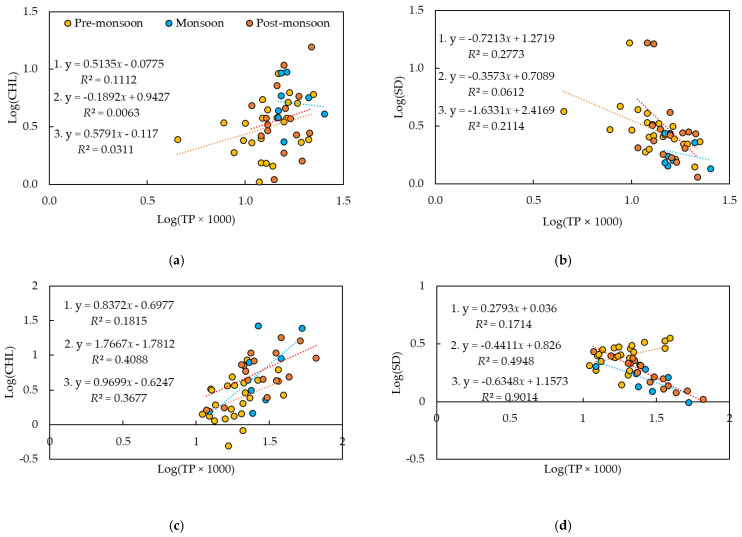

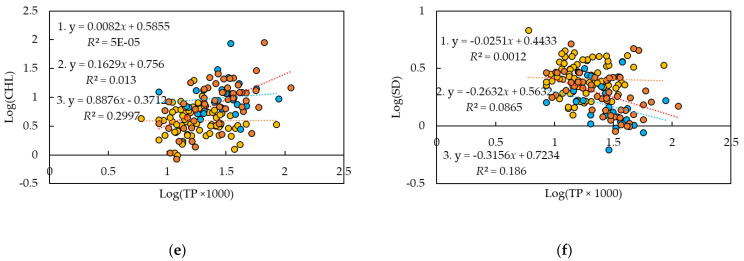
Empirical relationships between chlorophyll-*a* (Chl-*a*), Secchi depth (SD), and total phosphorus (TP) in the three precipitation year groups. (**a**) Log(Chl-*a*)-Log(TP × 1000) and (**b**) Log(SD)-Log(TP × 1000) in the drought years; (**c**) Log(Chl-*a*)-Log(TP × 1000) and (**d**) Log(SD)-Log(TP × 1000) in the flood years; and (**e**) Log(Chl-*a*)-Log(TP × 1000) and (**f**) Log(SD)-Log(TP × 1000) in the normal years. The results for the regression equations in the figures are: 1. Pre-monsoon season; 2. monsoon season; and 3. post-monsoon season.

As reported previously [7,68], TP appeared to be a better indicator of Chl-*a* than TN, the *R*^2^ values of which were less than 0.1, except for the monsoon period of the drought years (Figure 6). Chl-*a* and TP showed a noticeable correlation in the post-monsoon periods of the normal and the flood groups, with *R*^2^ values of 0.30 and 0.37, respectively. The drought years did not show any relationship with *R*^2^ ≤ 0.11 in each period. Interestingly, TN/TP did not show any appreciable relationship with Chl-*a* in all the periods of each group, with *R*^2^ values less than 0.10 except for the monsoon period in the flood years (*R*^2^ = 0.27), even though it was found to be a better indicator than TP in previous research [7]. This result was in agreement with the monthly concentration profiles of TP and Chl-*a* in the drought years (Figure 4).

The correlation of SD with TN was negligible except for the monsoon period of the flood years, when an *R*^2^ of 0.31 was identified (Table S1). This supported the fact that TN was mostly composed of TDN. TP showed a variable correlation with water clarity in different groups. Only weak correlations were observed in the pre and post-monsoon seasons in the drought years. The flood years, however, showed an appreciable correlation in the monsoon season (*R*^2^ = 0.49), and a very strong correlation in the post-monsoon season (*R*^2^ = 0.90). The normal years did not show much relationship between SD and TP. Chl-*a* distribution was better explained in the normal years (*R*^2^ = 0.31) than in the other two.

The results from the Pearson correlation and empirical regression analyses all indicated that the statistical relationships between water quality and hydrological parameters were significantly weaker than what was reported in a study performed over the same period (2000–2020) in the Daecheong Reservoir, a large reservoir located approximately 100 km north of the current study area [7]. The correlation coefficient values between the parameters that were related to particulate matters, such as TSS, TP, and TPP, which are expected to be washed from the watershed and contained in the runoff during the monsoon rainfall, were significantly lower. In addition, the results of the regression analysis indicated that precipitation and its related parameters, such as inflow and outflow, did not explain the variations in the levels of many parameters that are known to be strongly controlled by the duration and intensity of rainfall in a reservoir that is seriously affected by material input from its watershed.

The decoupling of the parameters in the study area in comparison with the case of the Daecheong Reservoir might have been caused by the differences in the drainage ratios between the two reservoirs. The drainage ratio is defined as the ratio of watershed area to water surface area of a reservoir, and is commonly used to identify the impact of the processes occurring in the watershed on the physical and chemical characteristics of a reservoir [69]. The ratio was utilized to identify the influences of the watershed on nitrogen removal [70], carbon input [71,72], and lake color [73]. It was reported that color change of a reservoir, a useful indicator of the concentration of dissolved allochthonous humic matter released from the watershed [74], showed a positive correlation with the drainage ratio [73]. The drainage ratio of Daecheong Reservoir was 44.0, with a water surface area and watershed area of 72.8 and 3204 km^2^, respectively, while the corresponding value for Okjeong Reservoir was 28.8. The low drainage ratio of the Okjeong Reservoir was anticipated, as it sits on the uppermost section of the Seomjin River, and it was also reported for the reservoirs in North America that water quality of lakes with a smaller watershed could be less sensitive to precipitation due to their tendency to have a longer residence time [62]. With a significantly lower ratio, it was expected that the impact of precipitation on water quality, which is primarily exerted in the form of transported dissolved and particulate materials contained in the runoff, would be significantly lower in the study area than in the Daecheong Reservoir.

### 3.7. Trophic State Index and Its Deviation

Temporal distributions of the annual TSI_C_, the arithmetic average of TSI_TP_, TSI_SD_, and TSI_Chl_, showed that the reservoir in general remained mesotrophic except for 2007, when a eutrophic condition with TSI_C_ = 51.8 was calculated (Figure S2). A consistent increase or decrease from the previous year was not observed in the years that belonged to the flood group. The drought year, however, showed appreciable decreases in 2008 and 2015, and low TSI_C_ values near the boundary between mesotrophic and oligotrophic conditions were maintained in the period from 2015 to 2019, when three drought years occurred. The most pronounced decrease in individual TSIs was observed with TSI_TP_ in each drought year. Evaluation of temporal TSI changes in different precipitation periods indicated that the most prominent decrease toward the end of the investigation period was observed in the monsoon period. Although the post-monsoon period also showed an observable variation, the pre-monsoon season did not show much change with time. The flood years did not show consistent changes in seasonal evaluation, even in the monsoon season. The drought years did show the largest decrease in TSI_TP_, followed by TSI_Chl_ and TSI_SD_. No significant variation was observed for TSI_TN_ in any seasonal evaluation.

**Figure 7:**
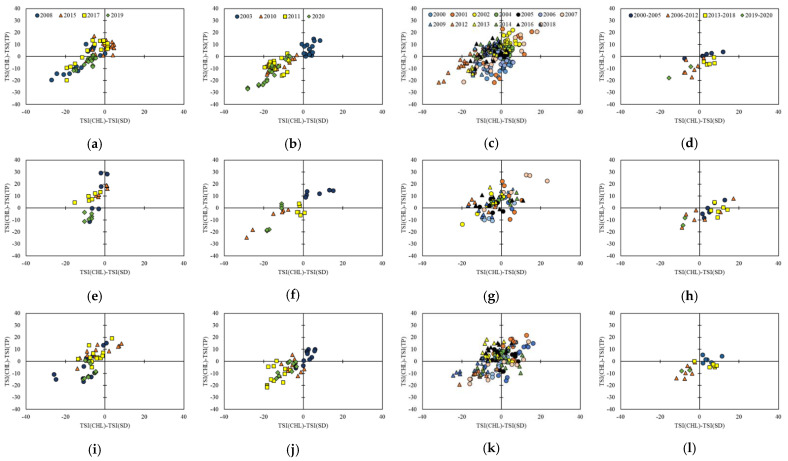
Variations in the trophic state index deviation (TSID): (**a**–**d**) represent the TSID in the pre-monsoon period of the drought, flood, and monsoon years, and the annual average values of the study period, respectively; while (**d**–**h**) show the monsoon period and (**i**–**l**) show the post-monsoon period using the same year group sequence as in (**a**–**d**).

An analysis of the limiting factors for the trophic status by identifying trophic state index deviation (TSID) using a two-dimensional graphical method based on the TSI_Chl_–TSI_TP_ and TSI_Chl_–TSI_SD_ relationships showed a pronounced difference in data distribution among the different precipitation groups (Figure 7). In the case of the normal precipitation group, the data were distributed rather evenly in the quadrants designating P-limited large particles (blue-green algae; the 1st quadrant), smaller particles including inorganic seston (2nd quadrant), and non-algal turbidity (3rd quadrant). Although a significant number of data were located in the area of zooplankton grazing (4th quadrant), the fraction occupied by them was relatively minor. The flood year group showed a similar distribution to the normal years, but the data in the 3rd quadrant occupied a dominant fraction, with only single data point identified in the 4th quadrant near the boundary to the 3rd quadrant. In addition, a significant decrease was observed in the 1st quadrant. Drought years showed a similar distribution to the flood years except for a larger fraction of data located in the 2nd quadrant. Normal years showed a relatively even distribution around the y-axis, but most data in the drought and flood years were located on the left-hand side, except for 2015 and 2003, indicating the dominating influence of small particles, not necessarily related to algae, on light attenuation [43]. This result was in accordance with the observation that the lowest Chl-*a* concentrations were observed in the drought years, and was reasonable, as inorganic particulate matter should be introduced in large quantities during the monsoon period of the flood years. Flood years also showed TSI_Chl_ < TSI_TP_ in most of the data, while the 2003 data showed the opposite.

Seasonal distributions of data in each group were also different. The normal precipitation group did not show much differentiation between the seasons, although zooplankton grazing was mainly observed in the pre- and post-monsoon periods. In the case of the flood year group, yearly differentiation was observed in each period, especially between the data for 2003 and the rest. Despite the fact that the strongest monsoon precipitation was recorded in 2003, almost all the data were located in the 1st quadrant, while the remainder were dominated by non-algal turbidity. In particular, the data from the year 2020 were almost exclusively located in the 3rd quadrant. The same trend was also observed in 2019, even though the year was classified as a drought year, with annual precipitation of 1069.7 mm. It appeared that P-limitation was alleviated from 2015 to 2019, as the change occurred mostly in the vertical direction, most likely due to an increasing trend of precipitation, with fluctuation from 2015 to 2019. An almost monotonic decline in Chl-*a* concentration was also observed in this period (Figure 3). This might have been caused by the changes in reservoir operation. Korean reservoirs typically reduce the water level in June to secure the volume that can accommodate the large influx of water during the monsoon season. Occurrence of a serious drought or lack of monsoon rain in the summer of 2015, however, adversely affected the operation method, and many of the reservoirs in the nation suffered a serious shortage of water. During the period, water levels in the reservoir in the monsoon and post-monsoon seasons generally increased with time (Figure S3), which may have caused the impact on TP, Chl-*a*, and SD [23,58].

Discrepancy in the data distribution among the years in the same precipitation group, especially between the 1st and the 2nd half of the investigation periods, implied the possibility that variation of trophic limiting factors might have been controlled by some factors other than annual precipitation. The average annual values of TSIs in each precipitation period were calculated over the entire study period, and were plotted (Figure 7) to test this possibility. The results showed that the TSID data were temporally grouped into four periods of 2000–2005, 2006–2012, 2013–2018, and 2019–2020, each displaying tightly packed data without serious variation among different precipitation periods. The grouping did not have any clear association with specific intensity or trend of precipitation, indicating the fact that trophic status of the reservoir was controlled by the factors that were not related to precipitation. Detailed further research is warranted in an effort to clearly identify the individual or combination of factors that determine the trophic variation.

### 3.8. Principal Component Analysis (PCA)

PCA was performed using water temperature, TSS, SD, Chl-*a*, TN, TP, N/P, BOD, COD, BOD/COD, EC, inflow, outflow, water storage, and precipitation as input variables on each precipitation group. Five principal components (PCs) were identified in the drought years explaining 71.5% of the total variance, four PCs in the flood years explaining 71.5%, and five in the normal years explaining 69.0% (Table S2).

For the drought years, PC1 showed a strong positive association with water temperature, TSS, outflow, and Chl-*a*, and a negative association with SD. Moderate positive correlations were observed with TP and inflow. PC2 showed a very strong association with TN and N/P ratio, moderate positive relations with BOD/COD and inflow, and a negative relation with TP. PC3 had a strong and moderate correlation with BOD and COD, respectively. PC1 of the flood years had a strong negative correlation with SD, and positive relations with TSS, water temperature, and COD. Moderate correlations with TP and the reciprocal of N/P were also observed. PC2 had strong correlations with inflow and outflow, and moderate correlations with precipitation and TN. PC3 was negatively associated with EC and N/P, and positively with TP and Chl-*a*. BOD and BOD/COD were strongly associated with water storage in PC4. PC1 of the normal years showed a very similar association with the factors to PC1. PC2 also was similar to the counterpart in the flood years, except for moderate association of TN. The same could be applied to PC3, but Chl-*a* showed a weak correlation. Overall, the patterns were very similar between the flood and normal years.

It is interesting that PC1 of each precipitation group showed strong or moderate correlations with loadings that were quite similar to each other. These loadings were mainly related to hydrological characteristics of water (WT and outflow) and particulate water quality parameters (TSS, TP, SD), which can be sensitively affected by the precipitation variation. PC2 in the drought years showed a positive correlation with the nutrient ratio, BOD/COD, and inflow, and a negative correlation with TP. These correlation patterns appeared to occur mostly in the pre-monsoon period, and seemed to indicate the possibility that some COD might be internally produced in the period [75]. PC2s of the drought and the flood years showed strong correlations with inflow, outflow, and precipitation, and appeared to indicate hydrological processes in association with strong monsoon rainfall. The similarity between the two groups seemed to support the observations of very similar concentration profiles of many water quality variables between them, and implied that overall dynamics of these parameters were more similar between these two groups, especially in response to the fluctuation of precipitation.

## 4. Conclusions

As opposed to the generally perceived view, variations in monsoon intensity did not have a decisive impact on the inter-annual dynamics of the water quality and trophic status of the Okjeong Reservoir over the period of 2000 to 2020. The drought and flood years did not demonstrate consistent behavior in the concentrations of many parameters that are known to be carried to the reservoir from the watershed in the form of runoff. The results of the TSID evaluation indicated that the changes in limiting factors that governed the light attenuation in the water body were not completely controlled by the precipitation pattern. Statistical analyses also resulted in significantly lower correlations between the hydrological and water quality parameters. This lack of impact of precipitation appeared to be caused by the relatively low drainage ratio of the study area, which reduced the influence of watershed processes on the dynamics of the reservoir.

Non-variant monthly distribution of BOD and COD, and an increase in concentration along the longitudinal flow direction, especially during the drought period, indicated that the impacts of watershed processes were not as critical as in the other reservoirs in Korea. A very high BOD/COD ratio indicated that a significant fraction of the organic matter was biodegradable, and the increase in the ratio within the reservoir seemed to insinuate the possibility of a significant contribution from autochthonous sources. Due to the nature of the monitoring data, however, direct evidence of internal contribution was not available at the time, and a detailed investigation of the distribution and characteristics of the sediments is required to confirm the actuality of this scenario.

Water quality management of reservoirs in Korea has been mainly focused on managing and reducing pollution sources in the watershed, based on the assumption that precipitation-induced runoff is the main source of pollutants present in the water. The results of this study, however, indicated that this management approach alone may not work universally for those reservoirs that are located in the uppermost section of the river and have a low drainage ratio, even if they are in the area under the influence of a monsoon climate. Physical characteristics of a reservoir, including location and drainage ratio, should be evaluated, and a monitoring system that can identify the presence of internal contribution of pollutants should be installed before determining the management policy to maintain good water quality in a reservoir. Spatiotemporal reservoir dynamics not completely controlled by precipitation, as well as the possibility of significant input from autochthonous sources (especially during the drought years), along with an increasing trend of climate fluctuation all over the globe, insinuate the need to devise an intra-reservoir water quality management policy in the study area.

## Figures and Tables

**Table 1:** Statistical data of water quality parameters, including the results of a long-term trend analysis based on a Mann–Kendall trend (MKT) test.

Parameters	S	*p*-Value	Minimum	Maximum	Mean	Stdev.	Sen’s Slope	Kendall’s Tau	Trend
Precipitation	−22	0.526	843.1	1974.2	1377.2	301.0	−7.560	−0.105	No trend
BOD	107	**0.001**	1.4	2.2	1.7	0.2	0.020	0.518	**Increasing**
COD	47	0.164	2.6	3.9	3.4	0.3	0.014	0.225	No trend
BOD/COD	70	**0.037**	0.43	0.56	0.50	0.04	0.003	0.333	**Increasing**
SS	19	0.586	2.2	4.4	3.2	0.6	0.015	0.091	No trend
TN	−12	0.740	1.4	2.1	1.8	0.2	−0.002	−0.057	No trend
TP	−96	**0.004**	0.012	0.041	0.024	0.007	−0.001	−0.462	**Decreasing**
TN/TP	66	**0.050**	58.0	106.0	92.8	11.4	0.687	0.314	**Increasing**
EC	149	**<0.0001**	85.8	162.8	124.0	25.1	3.522	0.711	**Increasing**
Chl-*a*	−90	**0.007**	2.8	17.5	6.9	3.2	−0.228	−0.429	**Decreasing**
SD	−30	0.381	1.4	2.7	2.1	0.3	−0.014	−0.143	No trend

**Table 2:** Pearson correlation analysis of water quality and hydrological parameters during 2000–2020.

	BOD	COD	B/C	TSS	TN	TP	TN/TP	WT	EC	Chl-*a*	SD	IF	OF	Prec.	TDN	TDP	TPN	TPP
BOD	1.0																	
COD	**0.50**	1.0																
B/C	**0.72**	−0.15	1.0															
TSS	0.31	**0.35**	0.12	1.0														
TN	0.22	0.24	0.11	0.21	1.0													
TP	0.09	0.27	−0.08	**0.52**	0.30	1.0												
TN/TP	0.04	−0.16	0.17	−0.30	0.18	**−0.64**	1.0											
WT	0.13	**0.37**	−0.11	**0.40**	0.20	**0.54**	−0.34	1.0										
EC	**0.36**	0.00	**0.42**	0.04	0.06	−0.32	0.26	−0.09	1.0									
Chl-*a*	0.05	0.18	−0.06	**0.41**	0.24	**0.41**	−0.16	0.25	−0.07	1.0								
SD	−0.10	−0.28	0.08	**−0.46**	−0.17	**−0.41**	0.32	**−0.50**	−0.08	−0.30	1.0							
IF	0.06	0.11	0.00	0.28	0.19	0.23	−0.08	**0.38**	0.06	0.10	−0.32	1.0						
OF	0.02	0.10	−0.05	0.19	0.21	0.25	−0.08	**0.47**	−0.03	0.13	−0.26	**0.72**	1.0					
Prec.	0.03	−0.04	0.05	0.05	−0.05	−0.05	0.02	−0.02	−0.02	0.04	0.05	0.06	−0.09	1.0				
TDN	0.19	0.04	0.20	0.17	**0.82**	0.23	0.27	0.17	0.15	0.17	−0.23	0.18	0.16	−0.04	1.0			
TDP	0.23	0.28	0.08	**0.43**	0.10	**0.69**	**−0.44**	**0.43**	−0.16	0.12	**−0.35**	0.18	0.24	−0.02	0.18	1.0		
TPN	0.16	0.23	0.03	0.10	**0.40**	0.19	−0.15	0.07	−0.07	0.15	0.00	0.09	0.09	−0.02	−0.20	−0.13	1.0	
TPP	−0.07	0.22	−0.22	**0.40**	**0.40**	**0.76**	**−0.57**	0.41	−0.31	**0.40**	−0.33	0.20	0.16	−0.08	0.15	0.04	0.38	1.0

Note: WT: water temperature; Prec.: precipitation; B/C: BOD/COD; IF: inflow; OF: outflow.

## Supplementary Materials

The following are available online at https://www.mdpi.com/article/10.3390/ijerph18168499/s1, Figure S1: Monthly inflow and outflow of Okjeong reservoir during (**a**) drought, (**b**) flood, and (**c**) normal precipitation years, Figure S2: Temporal variations of TSIs in the study area. Variations in (**a**) Total, (**b**) Pre-monsoon, (**c**) Monsoon, and (**d**) Post-monsoon periods, Figure S3: Monthly changes in water level of Okjeong Reservoir from 2015 to 2020, Table S1: Empirical relationships between water quality parameters in the three precipitation year groups, Table S2: Principal component analysis of water quality and hydrological parameters in drought, flood and normal precipitation groups.
