# Supplementary material for: Inter-Annual and Seasonal Variations of Water Quality and Trophic Status of a Reservoir with Fluctuating Monsoon Precipitation
Source: Int J Environ Res Public Health. 2021 Aug 11;18(16):8499. doi: 10.3390/ijerph18168499 (PMC8392102; doi:10.3390/ijerph18168499)
Supplement: Supplementary file 1 [file ijerph-18-08499-s001.zip › ijerph-1316690-supplementary.pdf]

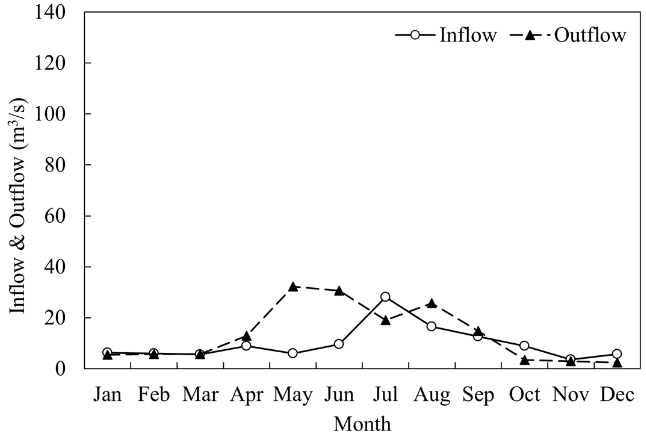

(a)

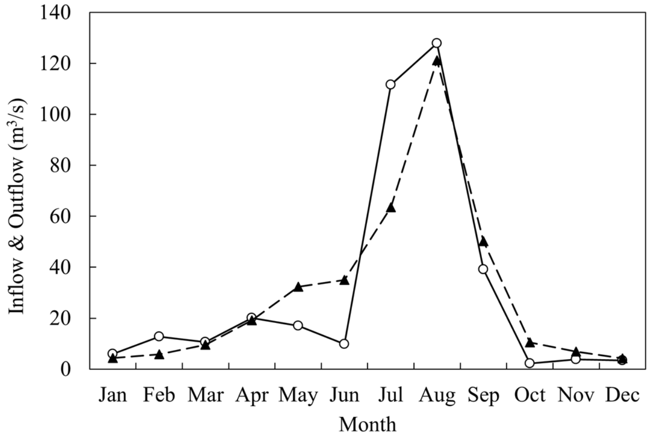

(b)

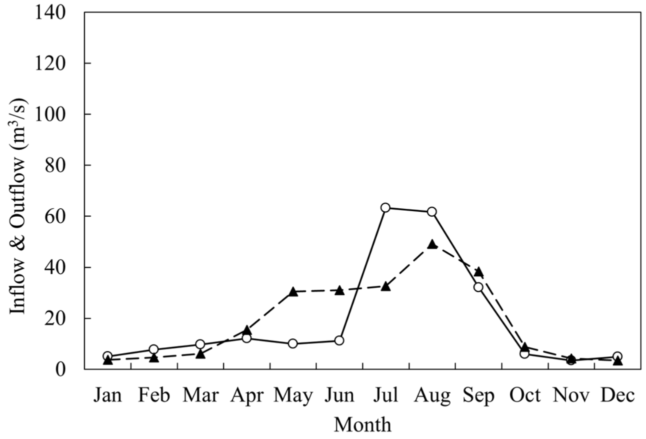

(c)

Figure S1. Monthly inflow and outflow of Okjeong reservoir during (a) drought, (b) flood, and (c) normal precipitation years.

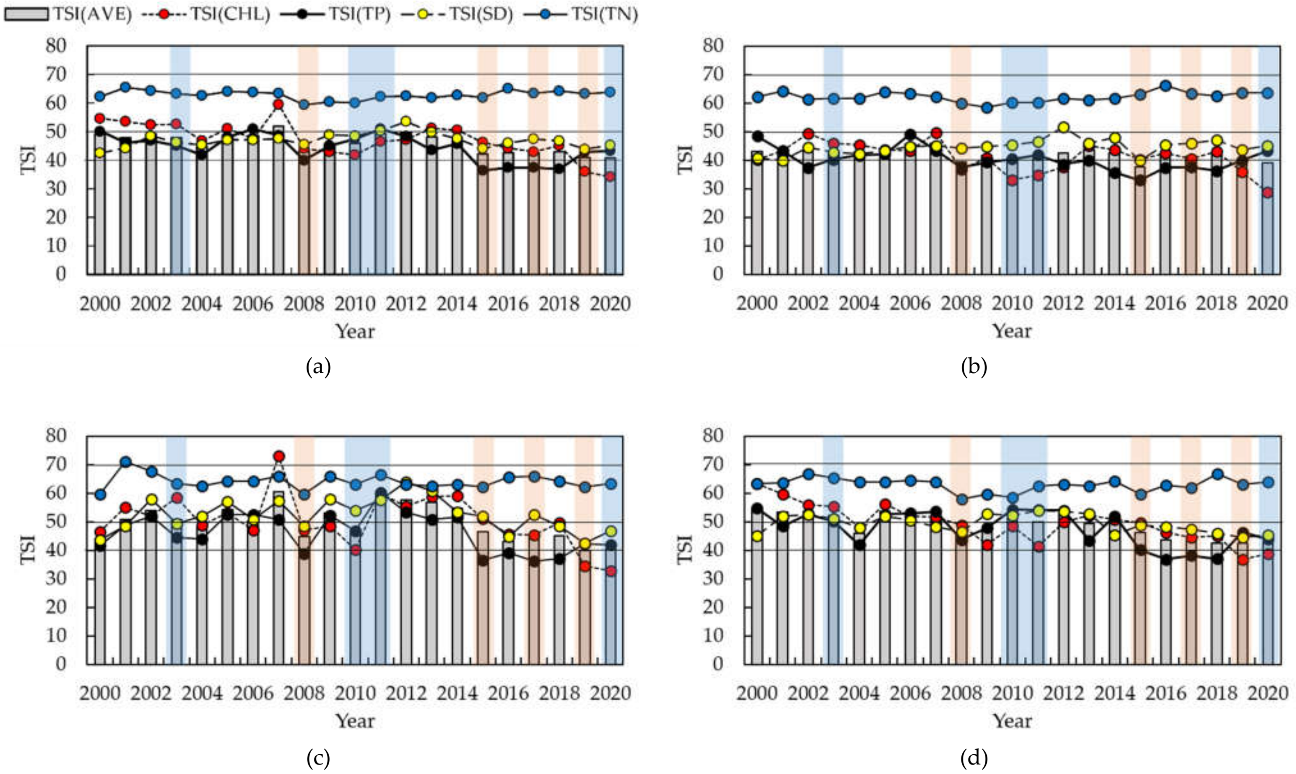

Figure S2. Temporal variations of TSIs in the study area. Variations in (a) Total, (b) Pre-monsoon, (c) Monsoon, and (d) Post-monsoon periods.

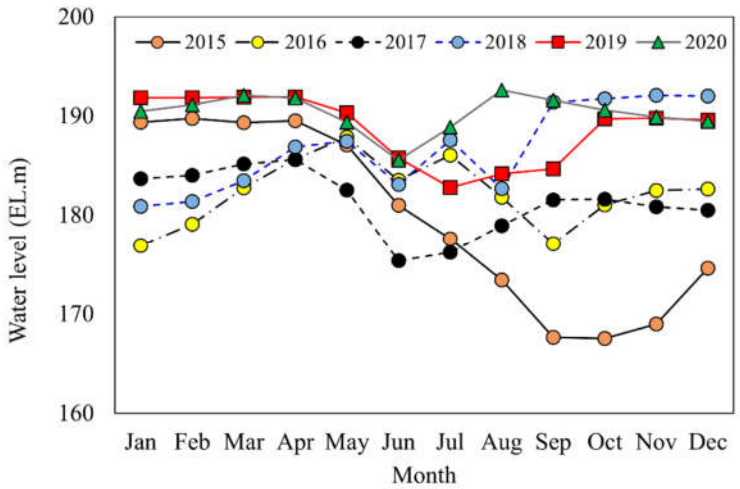

Figure S3. Monthly changes in water level of Okjeong Reservoir from 2015 to 2020.

Table S1. Empirical relationships between water quality parameters in the three precipitation year groups.

Drought years	Total			Pre-monsoon			Monsoon			Post-monsoon		
	R^2	a	b	R^2	a	b	R^2	a	b	R^2	a	b
Chl-TP	0.10	0.62	-0.16	0.11	0.51	-0.08	<0.01	-0.19	0.94	0.03	0.58	-0.11
Chl-TN	0.02	-0.41	0.64	0.09	-0.95	0.72	0.22	-1.03	0.95	<0.01	0.14	0.55
SD- Chl	0.16	-0.39	0.65	0.07	-0.24	0.59	0.25	-0.31	0.49	0.16	-0.44	0.72
TN-TP	0.08	-0.21	0.45	0.31	-0.26	0.53	0.02	0.14	0.06	0.02	0.17	-0.05
TN/TP-TP	**0.40**	-0.91	3.21	**0.59**	-0.89	3.22	0.07	-0.59	2.89	0.23	-0.71	2.86
TN/TP-TN	**0.46**	1.31	1.88	**0.45**	1.63	1.84	0.19	0.91	1.96	**0.45**	0.80	1.90
TN/TP- Chl	<0.01	-0.05	2.18	0.04	-0.16	2.31	0.09	0.29	1.96	0.04	0.08	1.97
SD-TP	0.21	-0.88	1.45	0.28	-0.72	1.27	0.06	-0.36	0.71	0.21	-1.63	2.42
SD-TN	<0.01	0.10	0.42	0.14	1.07	0.21	0.15	-0.51	0.39	<0.01	-0.08	0.48

Flood years	Total			Pre-monsoon			Monsoon			Post-monsoon		
	R^2	a	b	R^2	a	b	R^2	a	b	R^2	a	b
Chl-TP	**0.40**	1.33	-1.23	0.18	0.84	-0.70	**0.41**	1.77	-1.78	0.37	0.97	-0.62
Chl-TN	<0.01	-0.37	0.67	0.01	-0.41	0.48	0.04	2.00	0.11	0.03	-0.70	0.95
SD- Chl	0.14	-0.13	0.38	0.15	0.13	0.34	0.11	-0.08	0.25	0.24	-0.21	0.40
TN-TP	<0.01	0.02	0.24	0.08	0.14	0.09	0.10	0.08	0.20	0.05	-0.10	0.38
TN/TP-TP	**0.63**	-0.77	3.04	0.16	-0.23	2.37	**0.90**	-0.99	3.36	**0.77**	-0.98	3.29
TN/TP-TN	0.07	0.64	1.81	0.01	0.14	2.04	0.02	-0.50	2.11	0.26	1.37	1.53
TN/TP- Chl	0.25	-0.23	2.12	<0.01	0.00	2.08	0.27	-0.19	2.10	0.18	-0.30	2.10
SD-TP	0.27	-0.39	0.84	0.17	0.28	0.04	**0.49**	-0.44	0.83	**0.90**	-0.63	1.16
SD-TN	<0.01	-0.00	0.31	<0.01	-0.22	0.40	0.31	-1.31	0.61	0.07	0.43	0.14

Normal years	Total			Pre-monsoon			Monsoon			Post-monsoon		
	R^2	a	b	R^2	a	b	R^2	a	b	R^2	a	b
Chl-TP	0.16	0.61	-0.06	<0.01	0.01	0.59	0.01	0.16	0.76	0.30	0.89	-0.37
Chl-TN	0.03	0.72	0.57	<0.01	0.01	0.59	0.06	0.76	0.77	0.12	1.55	0.50
SD- Chl	0.31	-0.29	0.55	0.13	-0.21	0.54	0.12	-0.21	0.39	0.28	-0.24	0.49
TN-TP	0.01	0.04	0.21	0.01	0.04	0.22	<0.01	0.02	0.26	0.03	0.07	0.16
TN/TP-TP	**0.62**	-0.74	3.02	**0.55**	-0.67	2.96	**0.62**	-0.71	2.98	**0.58**	-0.72	2.96
TN/TP-TN	0.07	0.66	1.85	0.12	0.81	1.89	0.02	0.26	1.88	0.06	0.66	1.78
TN/TP- Chl	0.07	-0.17	2.16	<0.01	0.05	2.08	<0.01	-0.05	2.01	0.07	-0.16	2.08
SD-TP	0.13	-0.29	0.72	<0.01	-0.03	0.44	0.09	-0.26	0.56	0.19	-0.32	0.72
SD-TN	<0.01	-0.19	0.38	<0.01	0.11	0.38	0.05	-0.42	0.31	0.03	-0.35	0.36

Table S2. Principal component analysis of water quality and hydrological parameters in drought, flood and normal precipitation groups.

**Drought years**	**PC1**	**PC2**	**PC3**	**PC4**	**PC5**
Water temperature	**0.830**	-0.251	0.047	0.071	0.175
TSS	**0.742**	-0.248	-0.025	0.200	0.166
OF	**0.687**	0.002	0.102	-0.282	-0.133
SD	**-0.649**	-0.130	-0.057	0.342	0.035
Chl-*a*	**0.630**	-0.163	-0.029	0.217	-0.167
T-P	**0.587**	-0.419	0.319	0.005	0.133
IF	**0.527**	0.446	0.019	0.002	0.498
T-N	-0.133	**0.840**	0.205	0.031	0.003
T-N/T-P	-0.135	**0.825**	-0.236	-0.088	-0.012
BOD/COD	-0.312	**0.486**	0.370	0.458	0.119
BOD	0.085	0.053	**0.930**	0.145	-0.012
COD	0.447	-0.277	**0.662**	-0.189	-0.111
Discharge	-0.386	0.379	**0.553**	-0.404	-0.149
EC	0.032	-0.010	-0.002	**0.856**	-0.053
Precipitation	-0.041	-0.049	-0.074	-0.021	**0.913**
Eigenvalue	3.646	2.389	1.970	1.461	1.256
Percentage variance explained	24.307	15.928	13.133	9.739	8.370
Cumulative percentage variance explained	24.307	40.236	53.369	63.108	71.478

**Flood years**	**PC1**	**PC2**	**PC3**	**PC4**
SD	**-0.837**	-0.229	-0.150	0.036
TSS	**0.762**	0.176	0.197	0.085
Water temperature	**0.710**	0.479	0.065	0.212
COD	**0.695**	-0.434	-0.087	0.337
IF	0.121	**0.897**	0.127	0.133
OF	0.231	**0.826**	0.156	0.211
Precipitation	0.105	**0.610**	-0.082	-0.072
T-N	-0.081	**0.464**	-0.102	-0.261
EC	0.114	0.112	**-0.850**	0.148
T-P	0.501	0.068	**0.735**	0.004
T-N/T-P	-0.558	0.123	**-0.590**	-0.104
Chl-*a*	0.393	0.152	**0.583**	0.132
BOD/COD	0.054	0.073	-0.063	**0.904**
BOD	0.439	-0.253	-0.063	**0.826**
Discharge	-0.110	0.265	0.552	**0.642**
Eigenvalue	3.292	2.771	2.394	2.264
Percentage variance explained	21.948	18.472	15.962	15.093
Cumulative percentage variance explained	21.948	40.421	56.382	71.475

Table S2. Principal component analysis of water quality and hydrological parameters in drought, flood and normal precipitation groups (continued).

Normal years	PC1	PC2	PC3	PC4	PC5
COD	**0.827**	-0.174	-0.107	0.241	0.109
SD	**-0.705**	-0.327	0.063	0.150	0.083
Water temperature	**0.644**	0.459	0.069	-0.174	-0.082
Chl-*a*	**0.580**	-0.031	0.144	-0.060	-0.006
TSS	**0.560**	0.387	0.134	0.193	-0.120
IF	0.216	**0.812**	0.084	-0.104	0.172
Precipitation	-0.136	**0.735**	-0.050	0.232	-0.139
OF	0.441	**0.525**	0.194	-0.277	0.262
Discharge	0.058	-0.016	**0.800**	0.121	0.184
EC	0.198	-0.035	**-0.695**	0.347	-0.020
T-P	0.393	0.160	**0.627**	0.042	-0.298
BOD/COD	-0.255	0.098	-0.014	**0.845**	0.041
BOD	0.525	-0.088	-0.077	**0.791**	0.114
T-N	0.092	0.105	0.223	0.036	**0.826**
T-N/T-P	-0.292	-0.104	-0.483	0.129	**0.709**
Eigenvalue	3.137	2.040	1.912	1.787	1.480
Percentage variance explained	20.911	13.602	12.745	11.913	9.868
Cumulative percentage variance explained	20.911	34.513	47.258	59.171	69.039
